# Time‐dependent changes in bone healing capacity of scaphoid fractures and non‐unions

**DOI:** 10.1111/joa.12795

**Published:** 2018-02-27

**Authors:** Gernot Schmidle, Hannes Leonhard Ebner, Günter Klima, Kristian Pfaller, Josef Fritz, Romed Hoermann, Markus Gabl

**Affiliations:** ^1^ Department of Trauma Surgery Medical University Innsbruck Innsbruck Austria; ^2^ Division of Histology and Embryology Medical University Innsbruck Innsbruck Austria; ^3^ Department of Medical Statistics, Informatics and Health Economics Medical University Innsbruck Innsbruck Austria; ^4^ Division of Clinical and Functional Anatomy Medical University Innsbruck Innsbruck Austria

**Keywords:** scaphoid fracture, scaphoid non‐union, bone healing capacity, histological characterisation, time dependent changes

## Abstract

The scaphoid is the most frequently fractured carpal bone and prone to non‐union due to mechanical and biological factors. Whereas the importance of stability is well documented, the evaluation of biological activity is mostly limited to the assessment of vascularity. The purpose of this study was to select histological and immunocytochemical parameters that could be used to assess healing potential after scaphoid fractures and to correlate these findings with time intervals after fracture for the three parts of the scaphoid (distal, gap and proximal). Samples were taken during operative intervention in 33 patients with delayed or non‐union of the scaphoid. Haematoxylin and Eosin (HE), Azan, Toluidine, von Kossa and Tartrate‐resistant acid phosphatase (TRAP) staining were used to characterise the samples histologically. We determined distribution of collagen 1 and 2 by immunocytochemistry, and scanning electron microscopy (SEM) was used to investigate the ultrastructure. To analyse the samples, parameters for biological healing status were defined and grouped according to healing capacity in parameters with high, partial and little biological activity. These findings allowed scoring of biological healing capacity, and the ensuing results were correlated with different time intervals after fracture. The results showed reduced healing capacity over time, but not all parts of the scaphoid were affected in the same way. For the distal fragment, regression analysis showed a statistically significant correlation between summarised healing activity scores and time from initial fracture (*r* = −0.427, *P* = 0.026) and decreasing healing activity for the gap region (*r* = −0.339, *P* = 0.090). In contrast, the analyses of the proximal parts for all patients did not show a correlation (*r* = 0.008, *P* = 0.969) or a decrease in healing capacity, with reduced healing capacity already at early stages. The histological and immunocytochemical characterisation of scaphoid non‐unions (SNUs) and the scoring of healing parameters make it possible to analyse the healing capacity of SNUs at certain time points. This information is important as it can assist the surgeon in the selection of the most appropriate SNU treatment.

## Introduction

The scaphoid bone plays a critical role in carpal kinematics due to its complex three‐dimensional structure and function as a bridging link between the two carpal rows. Even a small fracture gap of 1 mm and angular deformities can negatively influence fracture healing (Herbert & Fisher, 1984; Krimmer et al. 2000). A total of 80% of the scaphoid is covered by cartilage, resulting in tenuous blood supply further increasing the risk of non‐union formation (Slade et al. 2003; Taljanovic et al. 2012).

The potential long‐term consequence of an untreated scaphoid non‐union (SNU) is severe osteoarthritis and pain (Krasin et al. 2001; Kawamura & Chung, 2008). Persistent SNU results in posttraumatic osteoarthritis in 75–97% of cases after 5 years and in 100% of cases after 10 years (Ruby et al. 1985; Inoue & Sakuma, 1996). For wrists with substantial degenerative changes of a scaphoid non‐union advanced collapse (SNAC), salvage procedures remain the only treatment option.

In the literature, there are several methods described to achieve anatomic reconstruction and bone fusion in SNU. They are based on the principle of restoring anatomy by correcting the deformity, providing stability, viability and vascularity (Slade & Dodds, 2006).

Depending on the chosen methods, union rates of up to 90% can be achieved. Vascular supply, structural bone properties, biological healing activity, and the time between initial fracture and intervention are important factors for successful surgical SNU treatment (Schuind et al. 1999).

The knowledge of bone structure and biology around the SNU therefore seems to be of great importance. In most studies, histology consists only of simple overview staining such as Haematoxylin & Eosin (HE) (Bervian et al. 2015). However, this does not sufficiently characterise the tissue under investigation. There is still a big gap in the literature concerning histological and immunocytochemical characterisation to interpret the biological state of SNUs. Further studies are therefore required to optimise diagnosis and therapy (Kawamura & Chung, 2008).

The present study aimed to select a set of histological and immunocytochemical parameters to describe the healing capacity after scaphoid fractures. These findings were then correlated with the time intervals after fracture and to the anatomical regions (distal fragment, gap and proximal fragment).

## Methods

### Patients

The study included 33 adult patients (28 male, five female) who were operated for delayed union or non‐union of the scaphoid without previous surgical interventions. Institutional review board approval and informed consent was obtained from all patients. The time between fracture and surgical intervention was recorded and the patients were divided into four groups according to the time passed from the initial fracture: 0–5 months (delayed union, five patients), 6–18 months (early non‐union, eight patients), 19–36 months (late non‐union, eight patients) and more than 37 months (old non‐union, 12 patients).

### Sampling

Samples were taken during surgical intervention adopting a volar approach and the SNU was excised completely. To avoid crushing the samples, we used a beaver blade (Beaver® 6900 Mini‐Blade®, Beaver‐Visitec International Heidelberg) or sharp chisels, which allowed for a precise en bloc resection. The sample size spanned the entire width and depth of the scaphoid reaching the dorsal, radial and ulnar cortical borders. In length, it reached at least 5 mm into the distal fragment and to the border of the cartilage at the proximal fragment. In two patients who received a free vascularised osteochondral graft, the proximal fragment was resected in one piece. In three patients treated with a partial wrist fusion, the whole scaphoid was resected.

Samples were marked with a surgical yarn at the distal end to distinguish it from the proximal end and gap. If an extraction in one piece was not possible, samples were extracted in three parts (distal, gap, proximal) by the surgeon and collected in appropriately labelled vials. All samples were optically digitised and anonymised in our laboratory by a technician.

### Fixation and embedding

Samples were collected into an 8% NaCl solution at surgery, transferred to the laboratory and immediately fixed with freshly prepared 4% paraformaldehyde solution in 0.1 m phosphate buffer at a pH of 7.4 (Schmiedinger et al. 2013). With the exception of samples designated for von Kossa staining and scanning electron microscopy (SEM), all samples were decalcified in 3% ascorbic acid, dissolved in osmotically equilibrated water (0.15 m NaCl), dehydrated in an ascending ethanol series and processed for paraplast embedding. Sections of 9‐μm thickness were cut and mounted onto polysine‐coated glass microscope slides.

### Histology

Samples were analysed after embedding and cutting in neighbouring slices. For histological analysis, we used the most central parts of the samples, thereby reducing the amount of damaged tissue. We carried out HE, Azan, Toluidine and Tartrate‐resistant acid phosphatase (TRAP) staining to gain more histological information. Furthermore, we performed immunocytochemical characterisation of the cartilage composition by monitoring collagen 1 and 2 distribution and defined distinct parameters for the healing status dependent on the location of fracture. In three cases with partial wrist fusion, the whole scaphoid was resected and, in addition to the above‐mentioned histological analysis, the ultrastructure of the entire scaphoid bone was visualised by SEM and von Kossa staining. These samples of long‐standing non‐unions served as a reference for clinically non‐active tissue.

On paraplast‐embedded sections, HE, Toluidine and Azan staining was carried out according to standard protocols described in *Romeis Mikroskopische Technik*, 19^th^ edition (Mulisch et al. 2015). We performed TRAP staining as described by Blumer et al. (2012).

Von Kossa stain of non‐decalcified, unembedded whole samples was implemented after the fixation of samples with formaldehyde (pH 7.4) as described in the fixation and embedding section of the manuscript. Post‐fixation was performed with 100% methanol for 12 h, 90% methanol for 2.5 h, followed by 70% methanol for 2.5 h. The samples were stained with 10% silver nitrate for 2 min followed by three washing steps with deionised water. Developing was performed with a sodium‐carbonate‐formol mixture (25 mL of 37% formaldehyde dissolved in 75 mL deionized water supplemented with 135 mg sodium carbonate, followed by a further washing step of 10 min with tap water. The staining was fixed for 5 min with 2% sodium thiosulphate and rinsed again for 10 min with tap water. Images were taken after 8 h to avoid further darkening of the silver nitrate.

### Immunocytochemistry

To further characterise the collected tissue, collagen 1 (the prevalent collagen in bone) and collagen 2 (the predominant collagen in cartilage) were detected with antibodies against respective isotypes of collagen. We used a primary rabbit anti‐human type I collagen (L. Fischer, National Institutes of Health, Bethesda, MD, USA) antibody for collagen 1 and a rabbit anti‐human collagen type II antibody (Cedarlane, Canada; ref: CL50211AP) for collagen 2. In both cases, HRP conjugated goat anti‐rabbit IgG (Dako/Cytomation, Glostrup, Denmark; Cat. No. PO448) was used as a secondary antibody. Detection was performed with a DAB‐MAP Detection Kit (Ventana, Vienna, Austria) according the diaminobenzidine development method with copper enhancement described by the company, followed by a light counterstaining with haematoxylin for 5 min. All samples were embedded in Entellan (Merck KGaA, Darmstadt, Germany).

### Image analysis

Digital images of histological and immunocytochemical samples were taken using a Nikon Eclipse 80i microscope (Nikon GmbH, Vienna, Austria) with a Nikon DS‐Ri1 digital camera. The Nikon NIS‐Elements Br 3.2 imaging software was used for photo documentation. Images of von Kossa staining on whole samples were taken on a Leica M50 stereo microscope with a Leica DFC450C camera. Images were processed for brightness and contrast correction with Adobe photoshop CS6.

### Scanning electron microscopy

In three cases with partial wrist fusion, the whole scaphoid was resected and the ultrastructure of the entire scaphoid bone was visualised by SEM. For analysis, samples were fixed with 2.5% glutaraldehyde in 0.1 m phosphate buffer (pH 7.4) followed by 1 h post‐fixation in 1% osmium tetroxide, dehydration with ethanol and critical point drying in a Bal‐Tec CPD 030 critical point dryer (Balzers, Lichtenstein). Specimens were mounted with conductive carbon cement Leit‐C after Göcke (Plano GmbH, Wetzlar, Germany) on aluminium stubs sputter‐coated with 10 nm Au/Pd (Balzers) and examined on a Zeiss SEM (Gemini 982).

### Histological evaluation

The parameters for the characterisation of the samples were chosen according to the experience of our histological experts and to existing parameters in the literature (Jarry & Uhthoff, 1971; Uhthoff & Rahn, 1981; Rein et al. 2010; Bervian et al. 2015). Parameters were defined and grouped according to three activity levels (high, partial and little) as described in Table 1 and were evaluated for their presence. The parameters known as normal positive signs of bone healing were grouped as signs of high healing activity. Parameters that represent biological activity but are atypical for healing or fewer in number, were defined as signs of partial activity. Parameters negatively associated with bone healing and biological activity were defined as signs of little activity.

**Table 1:** Assessed healing parameters grouped by activity level and region

Activity level	Distal fragment	Gap	Proximal fragment
High	Cell density	Dense filled	Cell density
	Trabecular thickening	Vessels	Trabecular thickening
	Osteoid formation	Cell richness	Osteoid formation
	Trabecular spikes	Collagen type 1	Trabecular spikes
	Cell lines	Collagen type 2	Cell lines
	Blood cells/vessels		Blood cells/vessels
Partial	Few cells	Empty spaces	Few cells
	Anorganic, sclerosis	No vessels	Anorganic, sclerosis
	Intertrabecular cells	Loose connective tissue	Intertrabecular cells
	Blood cells	Synovial layer	Blood cells
	Blasts/clasts	Collagen type 1	Blasts/clasts
	Necrosis	Collagen type 2	Necrosis
	Cartilage formation	Cartilage formation	Cartilage formation
Little	Giant cells, cysts	Giant cells, cysts	Giant cells, cysts
	Spotty activity	Spotty activity	Spotty activity
	Sclerosis seal	Sclerosis seal	Sclerosis seal
	Dense sclerotic area	Dense sclerotic area	Dense sclerotic area
	Foamy calcite deposit	Cartilage formation	Foamy calcite deposit
	Osteoclast		Osteoclast

Histopathological signs of high bone healing activity included cell density and cell types, trabecular thickening, osteoid formation, the presence of trabecular spikes bordered by osteoblasts (cell lines), neovascularisation and the presence of collagen 1.

Histopathological signs for bone degeneration consisted of the presence of a sclerotic seal on the fracture site, a sclerotic area, few cells, the presence of cysts, the presence of osteoclasts or macrophages, and the presence of a ‘foam deposit’ observed in areas with advanced bone degradation.

Histopathological signs of structure and the consistency of tissue in the gap comprised the status of gap filling, presence of blood vessels, type and concomitance of collagens. All histological evaluations were performed blinded to the patient history by two experienced histologists (H.L.E. and G.K.).

### Scoring

The samples from each patient were subdivided into a proximal, distal and gap section. We analysed each section for the presence of the defined parameters for high, partial and little activity of bone healing. For parameters in the high activity group, every point (presence of parameter) was scored with 2 (as this is evaluated as being better than partial activity). Partial activity was given a score of 1 (as in this case some healing is still taking place). Little activity was awarded a score of –1 (as this has to be considered negative) (see Table 2).

**Table 2:** Healing activity score

Healing activity	Score	Number of parameters		
		Distal	Gap	Proximal
High	2 Points	6	5	6
Partial	1 Point	7	7	7
Little	−1 Point	6	5	6
Score range		−6 to 19	−5 to 17	−6 to 19

### Statistics

Descriptive statistics are expressed as mean values and standard deviations or absolute and relative frequencies. Pearson correlation coefficients and linear regression analyses were calculated to investigate the relationship between the age of the SNU and histological and immunocytochemical findings. A significance level of α = 0.05 (two‐sided) was used. Statistical analyses were performed using the statistical software package SPSS, version 20.0 (SPSS Inc., Chicago, IL, USA).

## Results

### Selected healing parameters

**Figure 1:**
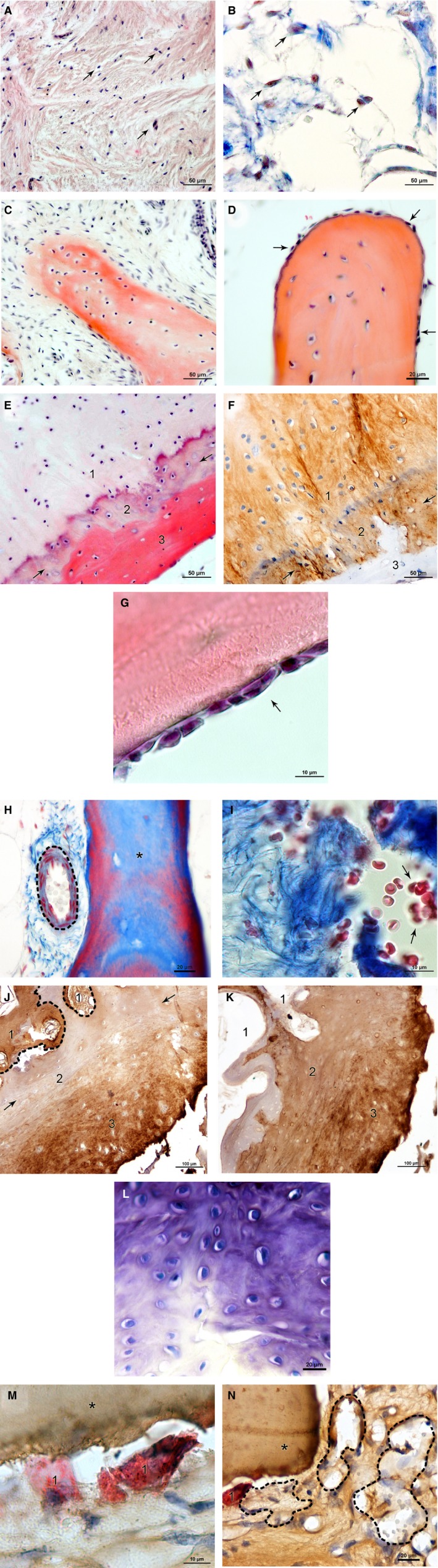
(A, B) Selected histological healing parameters in SNU samples: cell density, gap. (A) Connective tissue with densely packed cell nuclei (purple, arrows) and collagen fibres (pink) indicating highly active tissue; HE stain. (B) Less dense filled gap with fewer cell nuclei (red, arrows) and collagen fibres (blue) representing less active tissue; AZAN stain. Scale bars: 50 μm. (C, D) Selected histological healing parameters in SNU samples: trabecular spikes/thickening. (C) Trabecula formed by secreted extracellular matrix (red) and embedded osteocytes in typical spike formation; HE stain. (D) Osteoblasts (arrows) on the trabecular surface indicate trabecular thickening; HE stain. Scale bars: 50 μm (C), 20 μm (D). (E, F) Selected histological healing parameters in SNU samples: osteoid formation. (E) Cartilage calcification in the transition zone (2, arrows). Cartilage (1) transforms gradually into bone (3) with osteoid formation (red); HE stain. (F) Same sample with collagen 2 (brown) being present in the cartilage (1) and the transition zone (2), but not in bone (3); collagen 2. Scale bars: 50 μm. (G) Selected histological healing parameters in SNU samples: cell lines (osteoblasts). Lines of osteoblasts (purple, arrow) on the trabecular surface (red); HE stain. Scale bar:  10 μm. (H, I) Selected histological healing parameters in SNU samples: blood cells/vessels. Blood cells and blood vessels supply the tissue with nutrients, oxygen and precursor cells and are a prerequisite for healing. (H) Blood vessel (dashed line) next to a trabecula (asterisk); AZAN stain. (I) Blood cells (red, arrows) next to collagen fibres (blue); AZAN stain. Scale bars:  20 μm (H), 10 μm (I). (J,K) Selected histological healing parameters in SNU samples: collagen 1 (typical for bone) and collagen 2 (typical for cartilage), gap. The presence of both collagens was observed in some cases of SNU degradation. Predominance of collagen 2 next to the gap indicates formation of late SNU. (J) Collagen 1 (brown) is found in bone (dashed line, 1) and next to the gap (3) with unspecific cartilage stain in the middle portion (2); collagen 1. (K) The same sample shows collagen 2 (brown) in the middle portion (2) and next to the gap (3); collagen 2. Scale bars:  100 μm (J, K). (L) Selected histological healing parameters in SNU samples: cartilage formation, gap. Cartilage in the gap is a sign of encapsulation and SNU. Glycosaminoglycans (purple) and cell nuclei (blue) indicate the formation of cartilage; Toluidine stain. Scale bar: 20 μm. (M, N) Selected histological healing parameters in SNU samples: osteoclasts/foam deposits. High abundance of osteoclasts as well as foam deposits are a clear negative sign of bone healing capacity. (M) Osteoclasts (red, 1) next to a trabecula (asterisk); TRAP stain and collagen 1. (N) Foam deposits (dashed line) positive for collagen 1 (brown) next to an osteoclast (red, 1) and a trabecula (asterisk); TRAP stain and collagen 1. Scale bars: 10 μm (M), 20 μm (N).

Figure 1 provides an overview of selected healing parameters. Figure 1(A,B) shows a representative image of dense tissue (A) in contrast to less dense tissue (B). This parameter is especially important for healing capacity in the gap, as it indicates a high activity level for dense tissue, in contrast to ageing tissue with a reduction in cell density and numbers.

Figure 1 also shows trabecular thickening (C) and spike formation (D). These indicate formation of new bone by secreted extracellular matrix from osteoblasts, which leads to trabecular growth and finally to active healing. In Figure 1(E,F) new osteoid formation is visualised through the presence of a transition zone. This transition zone beneath the cartilaginous tissue can be seen in the HE staining by a purple layer and is positive for collagen 1 and collagen 2. Figure 1G is a typical example of cell lines (osteoblasts) indicating bone formation.

The presence of blood vessels and red blood cells shown in Figure 1(H,I) is a valid indicator of healing capacity, as it can be considered a prerequisite for adequate supplies of nutrients, oxygen and precursor cells.

Figure 11(J,K) shows the distribution of collagen 1, the predominant collagen in bone (J) and the distribution of collagen 2, the prevailing form in cartilage (K). Connective tissue is positive for both collagen forms, indicating a progressive SNU formation if collagen 2 is predominant in the gap.

Figure 1L indicates the presence of glucosaminoglycans as a sign of cartilage formation at the fracture site (gap). Similar to the presence of collagen 2, this implies the beginning of encapsulation and SNU formation.

Figure 1(M,N) shows an osteoclast and the related acidic resorption pit. The osteoclasts were visualised by TRAP staining, indicating the acidic phosphatases (M), and were often seen in the proximity of a collagen 1 positive foamy deposits (N). A high abundance of osteoclasts as well as deposits of foamy collagen 1 positive structures were negative signs of bone healing.

### Ultrastructure of an aged SNU

A control of ultrastructure in three cases of long‐standing SNUs was considered appropriate, as all parameters were assessed on decalcified samples. This provided an overview and detailed information on the surface and structure of a non‐decalcified proximal scaphoid pole sample.

#### SEM

**Figure 2:**
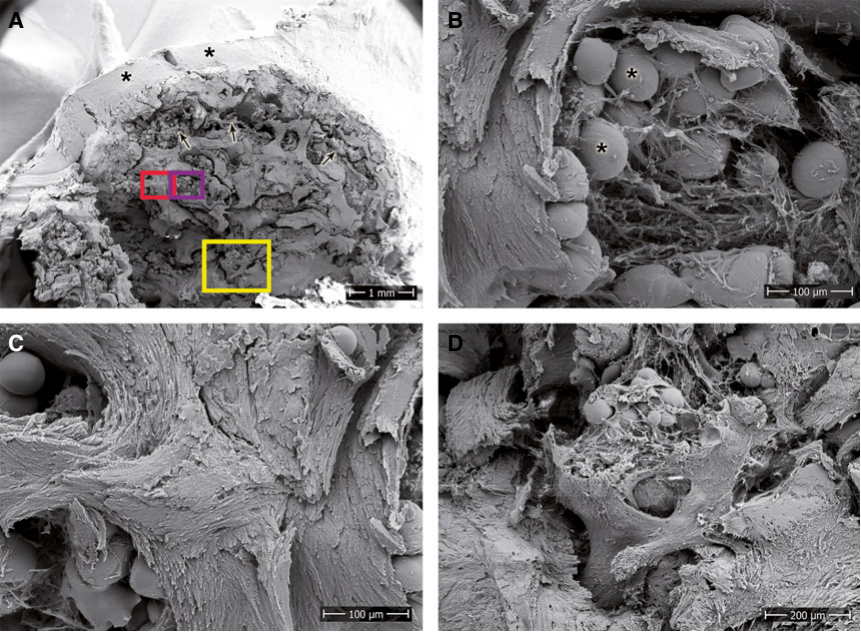
SEM of the proximal pole of a long‐standing SNU. (A) Overview image showing an outer cartilage layer (asterisks), abundant subchondral fat deposits (arrows) and an overall degeneration of the bone tissue. Rectangular areas highlight the location of the magnified images. (B) Fat deposits (asterisks) within loosely organised fibres of connective tissue. (C) Undirected orientation and (D) spongy degeneration of the trabeculae. Scale bars: 1 mm (A), 100 μm (B,C), 200 μm (D).

Figure 2A gives an overview of the proximal pole. The outer cartilage layer showed no abnormalities in cartilage ultrastructure. Beneath the cartilage layer an accumulation of fatty deposits was present (Fig. 2B). In summary, we observed an accumulation of branched ossified, trabecular structures of degenerative nature in the sample. The calcareous part of the bone seems to be the most affected. As seen in Fig. 2C, the microstructural orientation of the fibres was not aligned, as it should be in a healthy bone. Furthermore, parts of the trabeculae showed signs of spongy degeneration (Fig. 2D).

#### Von Kossa

**Figure 3:**
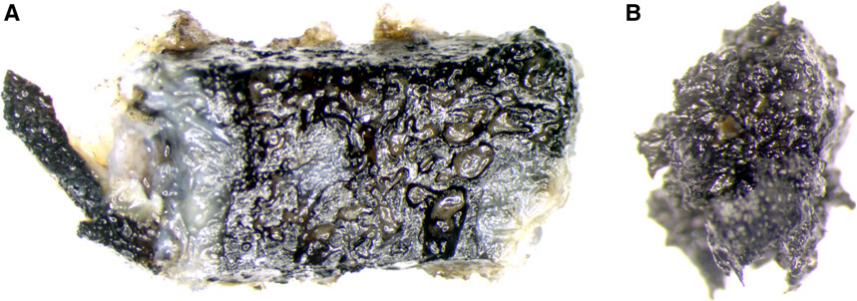
Von Kossa stain of a whole unembedded sample shows (A) trabecular structure intact at the distal part of the scaphoid bone and (B) a loss of the trabecular structure at the proximal pole from the same patient.

Figure 3 shows a von Kossa staining of a non‐decalcified tissue of a patient in the old SNU group. We performed the stain on the entire sample to emphasise that the decalcified histological samples we used for obtaining the parametric data for scoring, adequately showed a degradation of the calcareous parts of the bone. In the image, the distal part of this sample showed brighter intratrabecular structures compared with the more silver enhanced dark trabecular structure. A distinct trabecular structure is visible in Fig. 3A, whereas this feature was absent in the proximal part of the scaphoid (Fig. 3B), where a homogeneous dark mass with undistinguishable trabecular structures was observed.

### Correlation healing parameters to time since fracture

#### Distal fragment

**Figure 4:**
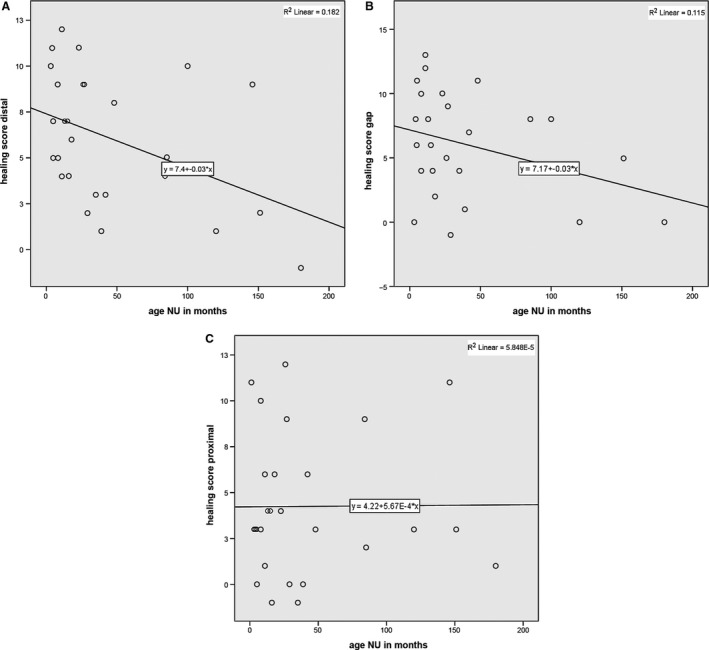
(A) Scatter plot of healing activity score of the distal fragment vs. time from initial fracture with regression line (y = 7.4 − 0.03*x) and *R* ^2^ (*R* ^2^ = 0.182). (B) Scatter plot of healing activity score of the gap section vs. time from initial fracture with regression line (y = 7.17 − 0.03*x) and *R* ^2^ (*R* ^2^ = 0.115). (C) Scatter plot of healing activity score of the proximal part vs. time since initial fracture; no correlation or change over time is visible.

The analysis of summarised healing activity scores of the distal fragment vs. the time from initial fracture, revealed a statistically significant correlation of *r* = −0.427 (*P* = 0.026). The slope of the line in a regression line showed a significant decrease of 0.029 scoring points per month (*P* = 0.026). Thus, the estimated average score at time zero was 7.4 (of a maximum achievable 19 points) and decreased to 2.1 at 15 years after initial fracture (Fig. 4A). This confirms the validity of the selected parameters and the decreased healing capacity of the fracture with increasing time from injury to surgery.

#### Gap region

For the gap section, findings were similar. There was a negative correlation between healing capacity score and time from initial fracture (*r* = −0.339, *P* = 0.090), with an estimated decrease of 0.029 scoring points per month (*P* = 0.090). The estimated score dropped from 7.2 (of a maximum achievable 17 points) at time zero to 2.0 at 15 years after initial fracture (Fig. 4B).

#### Proximal fragment

In contrast, the analyses of the proximal parts did not show a correlation for any patient (*r* = 0.008, *P* = 0.969) or a decrease in healing capacity over time according to the predefined parameters (Fig. 4C). Reduced healing capacity was already observed at the proximal part in samples collected at an early time point and the estimated score remained constant at a value of 4.2 over time (of a maximum achievable 19 points).

**Figure 5:**
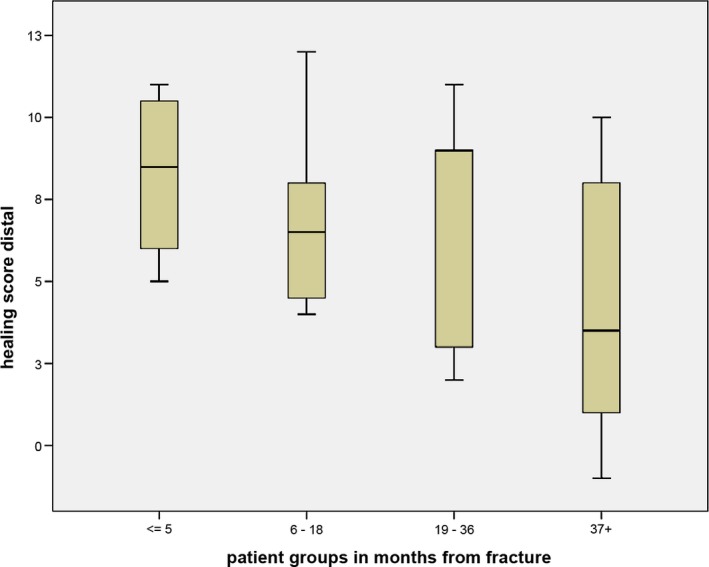
Box plot of healing activity score from the distal pole grouped by time since fracture. Central lines in each box denote the median, the lower/upper rims represent first/third quartiles and the whiskers extend to the minimum/maximum values.

By dividing the patients into four groups according to time passed since the initial fracture, the decreased healing capacity with increasing time becomes even more evident for the distal part (Fig. 5). The plots with grouped fracture age are not shown for the gap and the proximal part, as there was no significant relationship.

## Discussion

Many different surgical procedures have been proposed to achieve reconstruction and bone healing in SNU (Russe, 1960; Fernandez, 1990; Slade & Dodds, 2006). They differ in complexity and invasiveness for the patients. In the case of an avascular proximal fracture fragment, vascularised bone grafting is considered to be the gold standard (Gabl et al. 1999). This is in line with findings of Ribak et al. (2010) who showed that vascularised grafts have a better functional and fusion outcome compared with non‐vascularised grafts. For the clinician, it is important to apply the least invasive method that is still able to achieve bone healing. As the surgical procedures for treatment are manifold, the surgeon needs, in addition to refined radiological imaging (Schmidle et al. 2014), as much biological information on healing capacity as possible to make an informed decision.

In this study, we aimed to define a set of histological parameters that provides a comprehensive insight into the overall biological status of the tissue at a certain time point. First attempts to accomplish this have already been made (Rein et al. 2010; Bervian et al. 2015). However, histology in these publications is based primarily on overview staining. On this basis alone, no satisfactory qualitative tissue characterisation can be made. Parameters that have been used to characterise and classify SNUs are fibrous tissue in the ‘gap’, sclerotic closure of the non‐union (Herbert & Fisher, 1984), cystic changes at the fracture edges and osteoclastic resorption (Rein et al. 2010). Bervian et al. (2015) subdivided the SNU into four groups according to the vascularity, presence and appearance of osteoblasts, and the loss of trabecular regularity. In addition to these parameters, we assessed cell density, osteoid formation, the presence of trabecular spikes, cartilage formation and intertrabecular cells in order to assign a healing status of high activity, partial activity or little activity to the tissue. Furthermore, we assessed the presence of osteoclasts and a foamy calcite deposit as obvious signs of little activity. We also investigated whether collagen 1 and collagen 2 were detectable. As expected, we found an expression of collagen 1 in bone. In contrast, we found collagen 2 in the cartilage covering the scaphoid, and on occasion, in the material in the interstitial space and the connective tissue. By assessing the presence of these parameters, we were able to calculate a healing activity score for the distal fragment, the gap and the proximal fragment in all patients.

### Healing parameters

An early sign of a necrotic bone is reduced cell density in the gap showing the grade of gap closure. This, if present, indicates delayed healing. Osteoid formation, the formation of yet unmineralised matrix by osteoblasts, was considered a positive sign for vitality and was assessed by HE, Azan and collagen staining.

The presence of trabecular spikes with lining osteoblasts and trabecular thickening indicates new bone formation. In contrast, cartilage formation at the gap was considered a sign of SNU formation because a non‐union closed by cartilage was considered to be the beginning of encapsulation. The presence of few intertrabecular cells is a further sign of only partial activity in the fragments investigated. In late stages of SNU, we frequently found TRAP‐positive cells, specifically osteoclasts, indicating an increased tendency of bone resorption. A detailed description of bone repair and formation was presented by Shapiro (2008).

Given the shape of the scaphoid and the retrograde blood supply from distal to proximal, the absence or presence of blood vessels in each fragment can be seen as the basis for nutrient and oxygen supply of the tissue. Repair mechanisms, which are a prerequisite for active healing, require circulating precursor cells and healing factors/proteins. Bone has to be considered an organ with high self‐renewing capacity, with cell types such as osteoclasts and osteoblasts as well as healing factors such as bone morphogenetic proteins (BMP) playing a fundamental role (Phillips, 2005; Shapiro, 2008).

The equilibrium of bone resorption and bone formation is the fundamental mechanism of bone homeostasis. A single parameter is not able to depict this equilibrium of influencing factors. To display the healing capacity, we therefore pooled the individual parameters and scored them according to their level of biological activity. The parameters in the high activity group are normal positive signs of bone healing and were given a score of 2 points. Parameters in the partial activity group are atypical for healing or (in a semiquantitative assessment) fewer in number than in the high activity group. As they represent biological activity, they were scored positively with 1 point. Parameters in the group of little activity are negatively associated with bone healing and were accordingly scored with –1 point.

If this equilibrium is disturbed and bone resorption prevails, the bone lacks stability and integrity – as in the case of long‐standing non‐unions at the distal fragment, and to some extent at the gap region – leading to low healing capacity. At the proximal fragment, this equilibrium is disturbed irrespective of the time interval between injury and surgery.

In areas where osteoclasts were present, we found squamous tissue resembling at first glance remnants of fat cells. Upon closer inspection, however, the tissue stained highly positive for collagen 1. We referred to this tissue as foam deposit and characterised it as tissue with little activity. As a further control to ensure that this was calcified tissue, we performed a Von Kossa stain on an entire piece of bone from a ‘healthy’ distal pole and a ‘necrotic’ proximal one. As described in the Results section, we were able to demonstrate the presence of calcified material and the loss of structure in the necrotic part.

With SEM of a proximal pole, we were able to further characterise a late state of an SNU. As expected, the SEM clearly showed bone degradation with disorientation of collagen fibrils in the trabeculae, loss of trabecular structure, collapse of subchondral bone and formation of bone cysts, all of which are structural aspects that have been reported as characteristic for SNUs (Gabl et al. 1999; Paparo et al. 2012).

As decalcified bone was used for all of our histological investigations, the Von Kossa method and the SEM images were used as controls to validate the data collected from the parameters of histological images. They support our histological observation of bone degradation in late SNUs.

### Correlation healing parameters to time since fracture

For the surgeon, it is of crucial importance to treat the scaphoid fracture as soon as possible with the appropriate surgical method, as precarious blood supply and the high mobility of the bone increase the risk of SNU. The longer the period of non‐union, the poorer the outcome of anatomic reconstruction, regardless of the technique applied. This shows the dynamic nature of SNUs, which reduces their healing potential as time passes (Schuind et al. 1999). Other authors have also found a correlation between time intervals since fracture and healing capacity (Beutel & Wilhelm, 1999; Rein et al. 2010). In our study, we were able to score histological parameters that can be used to reflect the biological healing capacity in SNUs of up to 15 years.

For the distal fragment and the gap region, we were able to show an evident decline of bone healing activity over time. This is in line with clinical observations that the chances of successful healing decrease with increasing time from the fracture to the initial treatment (Schuind et al. 1999). In contrast to these studies, our histological investigations of the proximal pole showed that there was no correlation between positive healing activity and time. According to our data, the time elapsed since fracture is not a pivotal factor for the biological healing capacity of the proximal part. This is supported by a study of Boyer et al. (1998), who described, based on clinical observations, a generally decreased healing potential of the proximal pole in SNUs.

This study has shown the healing capacity for all three parts (proximal fragment, gap and distal fragment) of SNUs in a continuous course over time. Our results indicate that the time‐dependent decrease in healing capacity originates from the distal pole and gap region, whereas biological activity at the proximal pole does not change significantly over time and is low even at the early stages.

Non‐union is defined as the cessation of the periosteal and endosteal healing responses before union (Marsh, 1998). The patients were grouped in clinically relevant intervals according to the time passed since the initial fracture (Fig. 5). We found decreased healing capacity with increasing age between these groups at the distal pole. We did not expect a limited nutrient supply of the fractured distal poles, as they normally have better blood supply and are not subjected to a limited supply of nutrition, oxygen and circulating pre‐osteoblasts as is frequently the case for the proximal pole. This could be one of the reasons for the high healing score at early time points at the distal fragment.

For the proximal pole, there was no significant correlation between healing capacity and time. The score was already low for the early group, very similar to the score of the old group in the distal pole, suggesting that the healing capacity was poorer from early on after fracture in the proximal pole and remained poor over time. This shows that there is limited healing capacity of the proximal pole already at early stages with few alterations over time.

One limitation of our study is the low number of patients. However, the study still provides a thorough histological characterisation of scaphoid non‐unions over a long time range based on biological observations. We were able to establish a set of valuable parameters to describe the healing capacity of SNU. The results of this study may provide a histological basis for further clinical studies with an impact on patient treatment.

## Conflict of interest

The authors declare no conflicts of interest associated with this manuscript.

## Author contributions

Concept/design: G.S., H.L.E., G.K., G.M.; acquisition of data: G.S., H.L.E., G.M.; data analysis/interpretation: G.S., H.L.E., G.K., P.K., J.F., R.H.; drafting of the manuscript: G.S., H.L.E.; critical revision of the manuscript: G.S., H.L.E., G.M.; approval of the manuscript: G.S., H.L.E., G.K., P.K., J.F., R.H., G.M.
